# Development of Patient Derived Xenograft Models of Overt Spontaneous Breast Cancer Metastasis: A Cautionary Note

**DOI:** 10.1371/journal.pone.0158034

**Published:** 2016-06-29

**Authors:** Marta Paez-Ribes, Shan Man, Ping Xu, Robert S. Kerbel

**Affiliations:** 1 Biological Sciences Platform, Sunnybrook Research Institute, Toronto, Canada; 2 Dept. of Medical Biophysics, University of Toronto, Toronto, Canada; University of Alabama at Birmingham, UNITED STATES

## Abstract

Several approaches are being evaluated to improve the historically limited value of studying transplanted primary tumors derived by injection of cells from established cell lines for predicting subsequent cancer therapy outcomes in patients and clinical trials. These approaches include use of genetically engineered mouse models (GEMMs) of spontaneous tumors, or patient tumor tissue derived xenografts (PDXs). Almost all such therapy studies utilizing such models involve treatment of established primary tumors. An alternative approach we have developed involves transplanted human tumor xenografts derived from established cell lines to treat mice with overt visceral metastases after primary tumor resection. The rationale is to mimic the more challenging circumstance of treating patients with late stage metastatic disease. These metastatic models entail prior *in vivo* selection of heritable, phenotypically stable variants with increased aggressiveness for spontaneous metastasis; they were derived by orthotopic injection of tumor cells followed by primary tumor resection and serial selection of distant spontaneous metastases, from which variant cell lines having a more aggressive heritable metastatic phenotype were established. We attempted to adopt this strategy for breast cancer PDXs. We studied five breast cancer PDXs, with the emphasis on two, called HCI-001 and HCI-002, both derived from triple negative breast cancer patients. However significant technical obstacles were encountered. These include the inherent slow growth rates of PDXs, the rarity of overt spontaneous metastases (detected in only 3 of 144 mice), very high rates of tumor regrowths at the primary tumor resection site, the failure of the few human PDX metastases isolated to manifest a more aggressive metastatic phenotype upon re-transplantation into new hosts, and the formation of metastases which were derived from *de novo* mouse thymomas arising in aged SCID mice that we used for the experiments. We discuss several possible strategies that may be employed to overcome these limitations. Uncovering the basis of the failure to detect a high rate of overt spontaneous distant metastases having a heritable phenotype in PDX models may reveal new insights into the biology and treatment of advanced metastatic disease.

## Introduction

About a decade ago we began to develop preclinical models of advanced stage overt spontaneous metastasis of human tumor xenografts in immune suppressed mice for experimental therapeutics using established tumor cell lines [1–6]. The models now include breast cancer [1], malignant melanoma [2], ovarian carcinoma [3], colorectal carcinoma [4], and renal cell carcinoma [5]. The rationale for developing these models was that using them for *in vivo* therapy investigations would yield results having a better chance of predicting subsequent activity in patients, and hence clinical translation—at least with respect to the treatment of patients with metastatic disease, when compared to the more conventional approach of assessing drug activity based only on the response of established primary tumors [1]. An example of this, which we previously reported, is that treatment of SCID mice bearing established primary orthotopic breast cancer xenografts with one of the three different antiangiogenic drugs targeting the VEGF pathway, including the tyrosine kinase inhibitor (TKI) sunitinib, caused anti-tumor efficacy, whereas none of the drugs was effective in prolonging survival of mice with advanced metastatic disease [7] In addition combining sunitinib with standard chemotherapy did not improve chemotherapy efficacy in the advanced stage metastatic setting, whereas an antibody targeting the VEGF pathway was able to do so [7]. These results reflected the prior failure of multiple sunitinib based phase III trials in metastatic breast cancer, in contrast to the modest successes of bevacizumab plus chemotherapy in prolonging progression free survival [8–11]. The use of these new preclinical models of advanced metastasis has also been a factor in the decision to initiate multiple phase II and III low-dose metronomic chemotherapy clinical trials [12, 13], since certain metronomic chemotherapy regimens have been found to cause very potent efficacy effects even when treating mice with advanced visceral metastatic disease [1–3] despite in some cases showing minimal or no benefit when treating established primary tumors in control experiments [1, 2].

Also noteworthy is our finding that if prolongation of survival of mice with systemic metastatic melanoma can be achieved using a therapeutic intervention, a significant proportion of the mice relapse with spontaneous brain metastases [2], a clinically important phenomenon which is likely a manifestation of a brain ‘sanctuary’ phenomenon. In other words, clinically asymptomatic (occult) microscopic metastases in the brain, that are resistant to the therapy because of various possible factors, such as the blood-brain barrier, have more time to develop into symptomatic macroscopic metastases in the brain because of the temporary successful control of systemic disease [2].

We therefore decided to develop similar models of overt spontaneous metastasis using patient derived xenografts (PDXs), in this case, breast cancer PDXs. PDXs are being used increasingly to evaluate anti-cancer drug activity instead of human tumor xenografts derived from established cultured cell lines [14]. The rationale is that the cellular, molecular, and genetic properties of PDXs are highly similar to the original tumors removed from patient compared to long term established cell lines grown in tissue culture, and thus, in principle, should provide therapeutic results having a greater probability of clinical relevance and predictive translation [14]. However, therapy of PDXs almost always involves primary tumors, not distant overt metastatic disease. We reasoned treatment of metastases in PDX models may further improve their potential for predicting clinical activity, as well as providing an additional approach for studying the fundamental biology of metastatic disease.

We studied several breast cancer PDXs originally isolated and characterized by Welm and colleagues [15] to initiate these studies; we utilized a similar *in vivo* serial selection method adopted to successfully isolate variants with increased aggressiveness for spontaneous overt metastasis, not just micrometastasis after primary tumors resection, but using established cell lines—as described above and elsewhere [1, 2, 4, 5]. The methodology involved the following steps: i) orthotopic (intra-mammary fat pad) inoculation to increase the probability of spontaneous distant metastasis; ii) resection of established primary tumors within 3–4 weeks to prolong survival of mice, and thus more time for potentially seeded microscopic metastases to expand into overt lesions; iii) recovery of such spontaneous lung metastases which emerged after several months, and establishment of cell lines from them; iv) repeating the aforementioned three steps one more time [1, 2, 4, 5]. Using the established triple negative (TN) MDA-MB-231 human breast cancer cell line we noted that it could initially take 4–6 months for overt metastases (usually in the lungs) to form based on gross inspection, and these occurred in a majority of mice, after the second serial selection [1]. Moreover, the rate and extent of metastasis were accelerated and amplified during the second selection step, with spontaneous lung metastases occurring within one month after surgical resection of the primary tumors, and moreover, they occurred in a greater proportion of mice [1].

Here we report our experience using this approach employing several breast cancer PDXs, called HCI-001, HCI-002, HCI-004, HCI-008 and HCI-009 where we encountered several technical limitations that may restrict, or even preclude in some cases this approach as a practical strategy for studying metastasis of PDXs, at least using breast cancer PDXs, and thus utilizing such an approach for investigational therapeutics. These limitations include: i) an extremely low rate of distant overt metastases of human origin in SCID mice even after prolonged periods of observation; ii) very long latency periods for rare human metastases (in the lungs) to be detected; iii) emergence of spontaneous *de novo* tumors in the mice which upon analysis proved to be thymic lymphomas of mouse origin, spreading outside the thymus.

## Materials and Methods

All animal procedures, including maintenance and determination of experimental endpoints, were performed in strict accordance with the guidelines of the Sunnybrook Research Institute Animal Care Committee and the Canadian Council on Animal Care. The protocol was approved by the Animal Research Ethics Committee at Sunnybrook Research Institute. 144 female yellow fluorescent protein (YFP) severe combined immunodeficient (SCID) mice [16] were bred in house from breeding pairs originally and generously provided by Dr. Janusz Rak (McGill University, Montreal). Mice at 4–8 weeks of age were used. Several PDX tumors, as described previously [15], including the triple-negative (TN) HCI-001, HCI-002, HCI-004 and HCI-009 lines and the HER2+ HCI-008 line, were generously provided by Dr. Alana Welm (Huntsman Cancer Institute, University of Utah) and were propagated in YFP-SCID mice by serial passages; tumor tissue pieces 2–5 mm^3^, were implanted in the mammary fat pads of new animals, as described previously [1]. The take rate in all cases was 100%. Briefly, mice were anesthetized with isoflurane and a dose of buprenorphine (0.1 mg/kg) was administered (subcutaneously) and a 2 cm incision was made in the skin in order to expose the mammary fat pad. A 2–3 mm^3^ tumour piece was implanted orthotopically in the mammary fat pad and the skin was closed with wound clips. 24 hours after surgery another dose of buprenorphine was administered. The mice were daily monitored for any clinical signs (e.g. dyspnea, jaundice, hunched posture…) and weight and tumour size was recorded once a week. Mice were humanly killed by cervical dislocation when their tumor volume reached 1500 mm^3^ or they showed any clinical sign, as dyspnea, hind limb immobility, jaundice, body weight loss of 20% or presented a distended abdomen (a sign of ascitis).

Most of the experiments involved the use of the HCI-001 and HCI-002 PDXs. These were derived from primary tumors of two triple negative breast cancer patients [15]. The donor of the HCI-001 PDX had evidence of lung metastases at surgery while the donor of the HCI-002 had only lymph node metastases visible at the time of surgery [15]. The details of the origins and characteristics of the other three PDX lines are described in detail in Table 1 and the Online Methods of DeRose et al [15]. The 004 line was derived from a primary breast cancer from an infiltrating ductal carcinoma, 009 from the ascites of a patient with poorly differentiated adenocarcinoma and 008 from a pleural effusion of a patient with infiltrating ductal carcinoma [15]. Caliper measurements were carried out once a week to determine tumor growth and tumor volume was calculated using the formula *a*^*2*^ *b/2* where *a* is the width and *b* is the length. All tissue samples were originally collected with informed consent from individual being treated at the Hunstman Cancer Institute and the University of Utah under a protocol approved by the University of Utah Institutional Review Board, as noted in the Online Methods section of the study by DeRose et al [15].

Tumors were fixed in 10% buffered formalin and embedded in paraffin. Sections were stained with H&E. Antibodies used for specific tissue immunostaining included mouse monoclonal anti-HLA (1:200, Abcam), rat anti-mouse CD34 (1:100 LSBio), and rabbit anti-mouse Ki67 (1:100, Cell Signaling). The LSAB + HRP-system from Dako was used as a secondary antibody.

## Results

**Fig 1:**
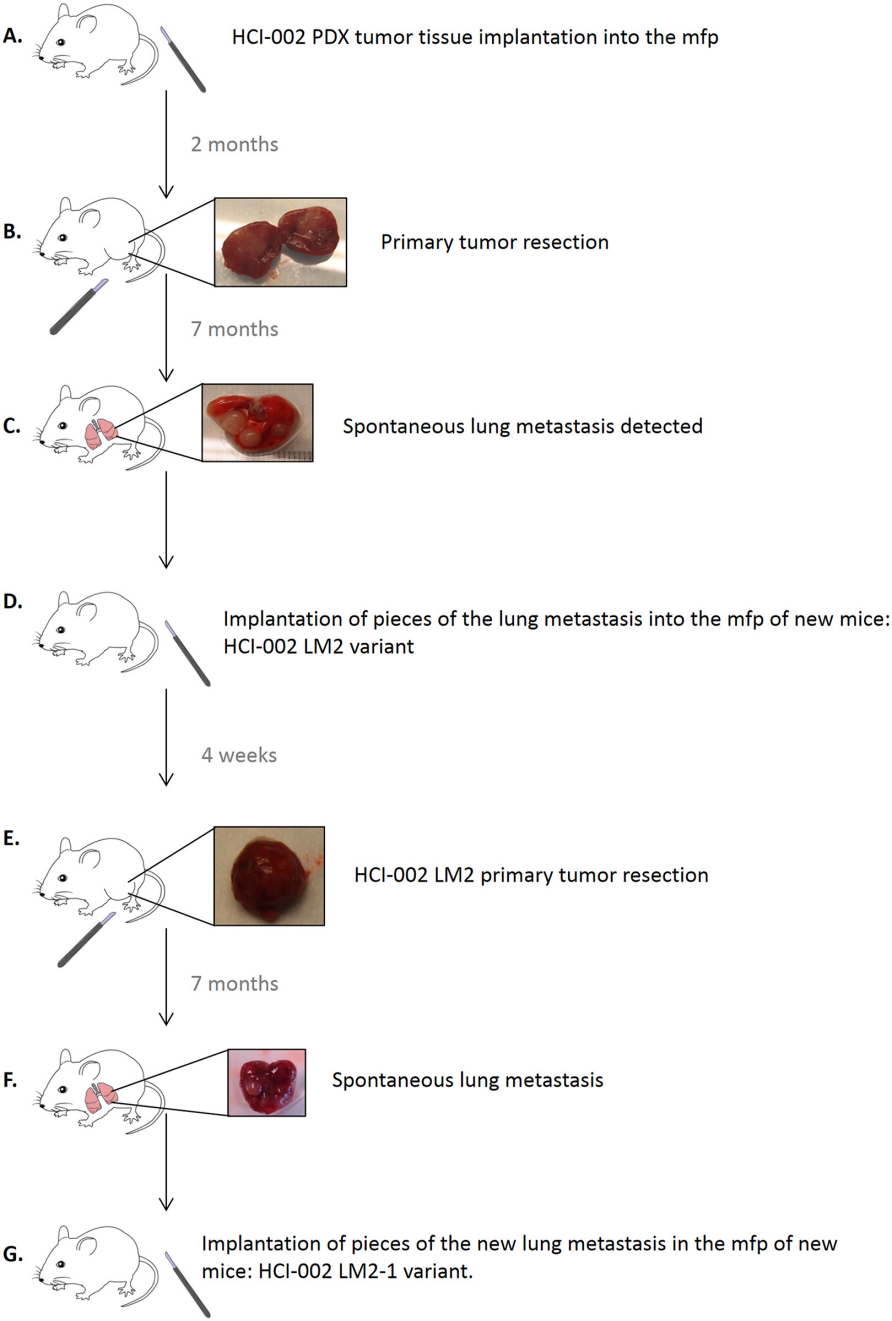
*In vivo* selection of a PDX breast cancer-derived metastatic variant. **A.** SCID mice were implanted in the mammary fat pad (mfp) with tumor fragments of the triple negative breast cancer PDX tumor HCI-002. **B.** Two months later, primary tumors were resected. **C.** After 7 months, one mouse developed on overt lung metastasis that was positive for the Human Leukocyte Antigen (HLA). **D.** Fragments of this lung metastasis were implanted in the mammary fat pads (mfp) of naive SCID mice and the variant called HCI-002 ML2 was isolated. This new variant grew with an accelerated rate when compared to the parental HCI-002 PDX. **E.** HCI-002 LM2 tumors were resected and mice kept alive. **F.** Seven months after primary tumor resection a spontaneous lung metastases was detected. **G.** Pieces of the metastatic nodule found in the lungs were implanted in the mfp of naive SCID mice, creating a new variant, called HCI-002 LM2-1.

**Fig 2:**
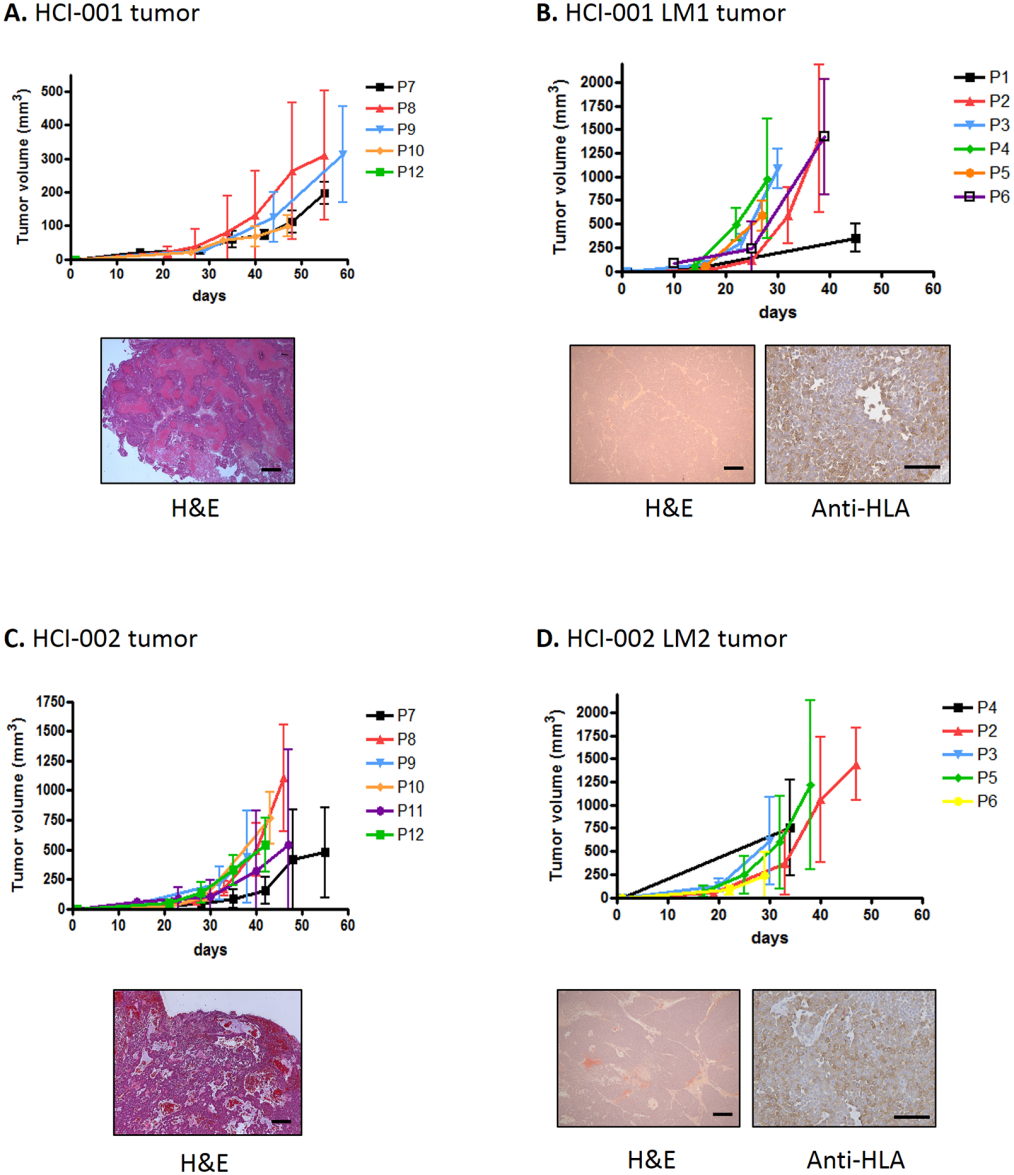
Acceleration of tumor growth of the variants derived from a metastasis. Graph showing the tumor growth rate of consecutive passages of two different tumors and their variants derived from lung metastases and representative slides stained for H&E: **A.** HCI-001; **B.** HCI-001 LM1; **C.** HCI-002; and **D.** HCI-002 LM2. Tumors did not show an increase in their growth rate with successive passages. The variants derived from lung metastases have an accelerated growth when implanted orthotopically as primary tumors in the mfp compared to the parental tumors. Scale bars, 150 μm.

In an attempt to develop a metastatic variant from several PDX breast tumors, tissue fragments from five different PDXs (HCI-001, HCI-002, HCI-004, HCI-008 and HCI-009 [15]) were first implanted in the mammary fat pads of SCID females. Fig 1 shows the step-by-step procedure of developing a variant from a metastatic nodule. When HCI-002 TN primary tumors reached 800 mm^3^, they were resected and mice observed afterwards. When a spontaneous metastasis of human origin developed (12 months after primary tumor implantation, and 10 months after tumor primary tumor resection) and could be detected by gross inspection., 2–5 mm^3^ pieces of the metastatic nodule (which only arose in the lungs) were implanted orthotopically in the mammary fat pad of 5 new SCID females, designating the new variant HCI-002 LM2. All 5 mice developed tumors in the mfp. When this variant, with an accelerated growth rate, developed spontaneous metastases large enough to be detected by gross morphology at time of necropsy (also after prolonged waiting periods), they were orthotopically implanted in the mammary fat pad of new SCID mouse recipients, and a new serial variant was designated, HCI-002 LM2-1, with the aim of developing a uniform and reliable metastatic tumor model. We focused our efforts mainly in the two TN PDX tumors that had more rapid growth rates: HCI-001 and HCI-002 (shown in Fig 1), both of which were originally derived from primary breast tumor tissue [15]. Many or even most of the mice developed tumor regrowths at the site of primary tumor resection: 20 mice implanted with HCI-001 (52%) and 47 mice implanted with HCI-002 for HCI-002 (78%). Mice were euthanized when such regrowths reached endpoint (a tumor volume of 1500 mm^3^). From the group of mice that did not manifest such tumor regrowth (19 implanted with HCI-001 and 13 implanted with HCI-002), and had longer survival times, only one from the HCI-001 PDX and two from the HCI-002 PDX, eventually developed lung metastases of human origin visible at naked eye at the time of necropsy and which could then be implanted orthotopically in new SCID females. As shown in Fig 2, successive passages of HCI-001 and HCI-002 tumors maintained a similar growth rate, and the tissue architecture of the tumor also remained stable. The tumor variants developed after implantation into the mammary fat pad of the metastatic nodules found in the lungs, had accelerated tumor growth rates, which were also maintained with successive passages. However the time needed to develop lung metastasis was not accelerated and was in fact similar to the parental tumors, an observation that stands in contrast to our previous results using established TN breast cancer cell line MDA-MB-231 [1].

**Fig 3:**
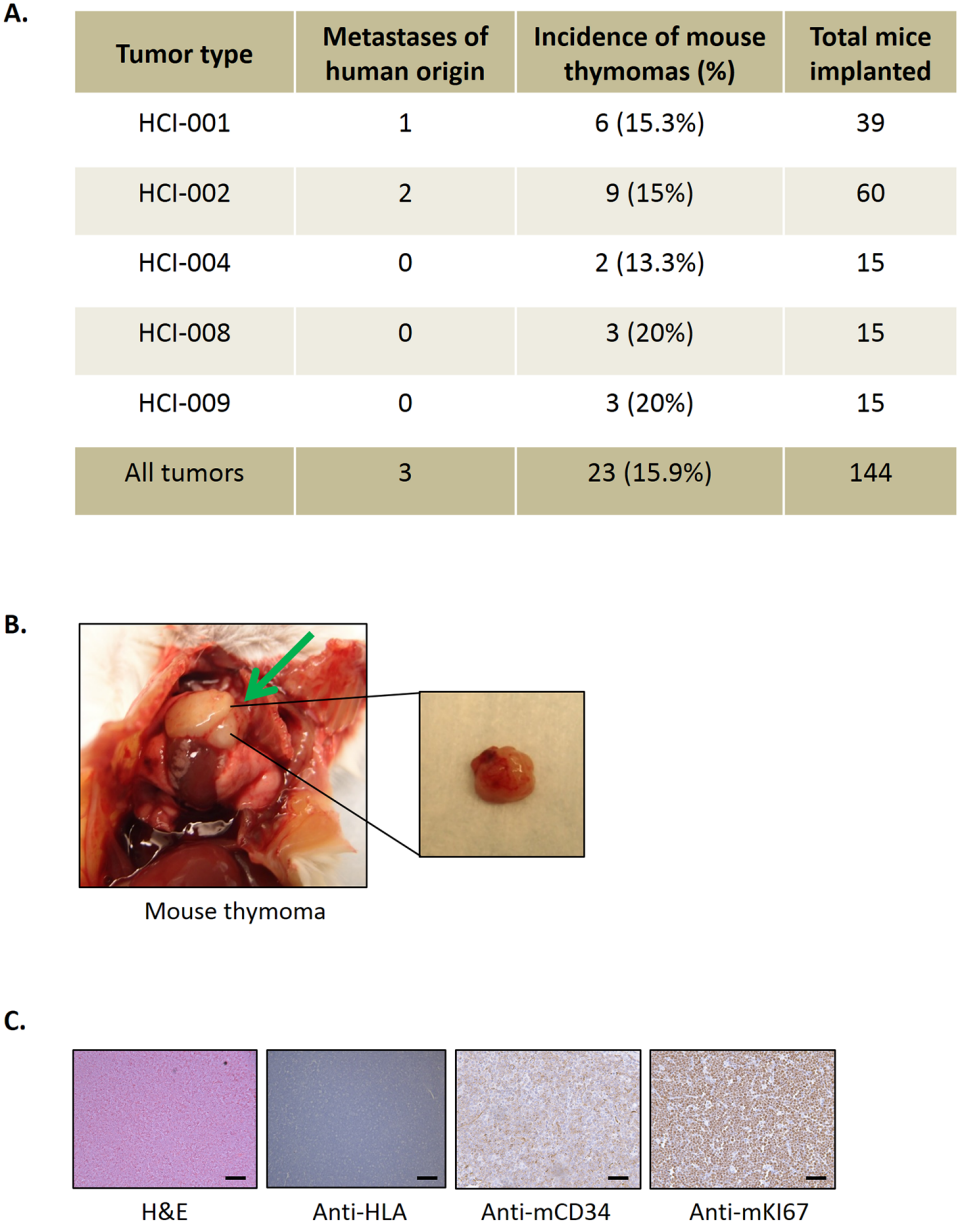
Increase in the percentage of mouse-derived thymomas. **A.** Table showing the numbers of metastases of human origin and the number of tumors of murine origin (mouse thymic lymphomas) found in all the tumor types. **B.** Gross morphology of a thymic lymphoma. The arrow shows a mouse thymoma. **C.** Histopathological sections of a thymic lymphoma of mouse origin stained with H&E, anti-HLA, anti-mouse CD34 and anti-mouse Ki67. These tumors (negative for HLA, with no human component) presented high rates of proliferation and were positive for the hematopoietic progenitor cell antigen CD34. Scale bars, 150 μm.

We also detected mouse-derived tumors (thymic lymphomas) when implanting PDXs in SCID mice that we had not previously encountered to such an apparent high degree when studying established cell lines. Fig 3A shows the number mice implanted with every tumor-type and the percentage of them which developed visible human lung metastases and mouse thymomas at time of necropsy, both human and mouse-derived. As has been previously described, SCID and NOD-SCID mice with age have a high incidence of spontaneous thymic lymphomas (Fig 3B) [17–19]. However, in our experience the incidence of such tumors seems to be much less common when implanting established cell lines. Thus, over the same time period, we detected 23 thymomas in the 144 mice implanted with five different types of breast PDX tumors (15.9%) whereas when using established cell lines (e.g. the metastatic variant LM2.4 [1]), the number of mice which developed thymomas was only 2 among 178 (1.1%). This apparently higher incidence is likely a result of studying PDX-bearing mice with a longer life span because of the slower growth rate of the PDXs, compared to mice injected with rapidly growing tumors established from injection of cells from established cell lines.

As shown in Fig 3C, these thymomas were negative for the expression of Human Leukocyte Antigen (HLA), meaning they had no human component, presented high rates of proliferation and they were positive for the hematopoietic progenitor cell antigen CD34.

## Discussion

Taken together our results highlight several potential issues to consider when trying to establishing post-surgical metastatic PDX models, which in this case apply to breast cancer PDXs in particular but which could conceivably be relevant to other types of PDXs. These include the slow tumor growth rates, and the unexpected rarity of spontaneous overt metastases of human origin as such metastases were observed in only 3 of 144 mice examined, and more specifically, in 3 out of 32 mice without evidence of tumor regrowth at the site of primary tumor resection. This extremely low rate of overt spontaneous metastasis was not expected given an impressive and interesting body of work by Hoffman and colleagues, much of it published two decades ago [20, 21]. These investigators assessed development of metastases, mostly in athymic nude mice by a procedure of “surgical orthotopic implantation” (SOI) of histologically intact tumor tissue fragments obtained from patients, and moreover, almost always without surgical resection of the primary orthotopic xenografts [20, 21]. These studies involved a broad range of cancer types such as gastric cancer [22], pancreatic cancer [23], cervical cancer [24], renal cell carcinoma [25], colorectal cancer [26], small cell lung cancer [27], among many others. Such models are currently called “PDOXs”–patient-derived orthotopic xenografts [21]. One of these older studies involved histologically intact human breast cancer tissue transplanted to the mammary fat pads of nude mice which resulted in the development of spontaneous lung metastases in 6 of 8 mice; in contrast, subcutaneous transplantation of the breast cancer tissue resulted in no detectable metastases in any of 7 mice evaluated [28]. In retrospect, this was the first orthotopic transplant metastatic model of human breast cancer. The tissue was obtained from a patient with poorly differentiated ductal breast cancer.

Another unexpected observation was the failure of spontaneous lung metastases of human origin arising in mice to manifest a more aggressive metastatic phenotype upon re-transplantation into new SCID mouse recipients. This stands in marked contrast to the results we have observed using established cell lines, as summarized in the Introduction and elsewhere [1, 2, 5, 6]. However, we were only able to test the 3 PDX-derived lung metastases in this regard. Another technical concern was the very high rate of regrowths of the site of primary tumor resection with limited duration of the survival time of such mice, although those high rates were also observed at the primary tumor resection site when using the established cell line such as MDA-MB-231 LM2-4 [1, 6]. Regrowths at the primary tumor resection site can also be a significant problem in patients with triple negative breast cancer [29–31]. In view of our results, there are several factors which might help explain the lack of detectable spontaneous metastases in our breast cancer PDX models and studies. These include the following: i) the results may have been influenced by the decision to study breast cancer PDXs. Thus, it is well known that the relative success of generating primary tumors using PDXs can vary significantly with the nature of the tumor type studied. Certain types of cancer such as melanoma and colorectal cancer generally have high take rates when patient derived tumor tissue is transplanted into immune suppressed mice, whereas other tumor types such as breast and prostate cancer have much lower tumorigenicity success rates [14]. Thus the same relative tumor-type dependency may also apply to ability to grow as distant spontaneous metastases. It will be of interest, in this regard, to evaluate colorectal carcinoma or melanoma liver metastasis development using the approaches we have described here, using colorectal cancer or melanoma PDXs. In this regard unpublished results by Dr. Catherine O’Brien (personal communication) also showed a complete failure to detect distant spontaneous metastases generated from colorectal cancer PDXs implanted orthotopically (in the cecum) of highly immune deprived NSG mice; moreover, these colorectal carcinomas were derived from both primary tumors and liver metastases; ii) The work by others including Hoffman and collaborators rarely involved an attempt to isolate spontaneous metastases and develop sublines from them to determine if they displayed a more aggressive and heritable spontaneous metastatic phenotype, and moreover, in a number of these studies it was mainly small microscopic metastases—not just overt metastases—that were detected; iii) we used SCID mice as the recipients for our studies. In contrast, use of mice with additional immune deficiencies such as NOD-SCID-IL-2γR-1^-/-^ (NSG/NOG) mice may result in a much greater level of distant spontaneous metastases developing at a more rapid rate. In this regard, we reported that the metastatic properties of a triple-negative breast cancer cell line such as MDA-MB-231, after surgical resection of orthotopically grown/transplanted primary tumors, was remarkably more aggressive for spontaneous metastasis formation in such mice compared to recipient control SCID or NOD-SCID mice [32]; iv) additional serial selections might also eventually result in subline variants with a greater rate and degree of spontaneous metastasis, as a specific PDX might be derived from a region of the tumor formed by subclones lacking metastatic potential [33, 34]; v) finally, most PDXs have been derived from primary tumors rather than distant metastases and thus it may be that a tissue specimen derived from a primary tumor—even if from a patient that subsequently developed metastatic disease—may contain a rarity of metastatically competent cells, and a different mutation spectrum, in comparison to a distant established metastasis derived from the primary tumor [35–37]. However, as noted above, the colorectal carcinoma experiments of Dr. Catherine O’Brien involved an assessment of PDXs derived from colorectal cancer liver metastases, not just primary tumors, and the PDXs were implanted into NOD-SCID-IL-2γR-1^-/-^ (NSG/NOG) mice.

When considering the possibility of developing metastatic PDX models for future studies, awareness of one or more the aforementioned factors might be considered as a pro-active strategy to improve the likelihood of generating distant spontaneous metastases in clinically relevant organ sites, in a practical manner, which would facilitate studies of the biology and treatment of human metastatic disease in an experimental mouse setting. Finally we would note that if our observation of failure or low success rate of developing PDX models showing evidence of overt spontaneous metastases can be reproduced, identifying the basis for the lack of such metastases, despite robust growth as primary tumors, may reveal valuable new insights into the biological basis and control of metastases, as well as treatment of metastatic disease.
